# Differential Expression of Inflammatory Markers in Hypoglycemia Unawareness Associated with Type 1 Diabetes: A Case Report

**DOI:** 10.3390/brainsci11010017

**Published:** 2020-12-25

**Authors:** Yousef Al Zoubi, Bashair M. Mussa, Ankita Srivastava, Abdul Khader Mohammed, Elamin Abdelgadir, Alaaeldin Bashier, Fatheya Al Awadi, Salah Abusnana

**Affiliations:** 1 Sharjah Institute for Medical Research, University of Sharjah, Sharjah 27272, UAE; yousef7orani-1996@outlook.com (Y.A.Z.); ankita2112@gmail.com (A.S.); amohammed@sharjah.ac.ae (A.K.M.); 2 Basic Medical Sciences Department, College of Medicine, University of Sharjah, Sharjah 27272, UAE; 3 Dubai Health Authority, Dubai Hospital, Dubai 4545, UAE; alaminibrahim@hotmail.com (E.A.); alaaeldin11@gmail.com (A.B.); alawadi1122@hotmail.com (F.A.A.); 4 Diabetes and Endocrinology Department, University Hospital Sharjah, Sharjah 72772, UAE; salah.abusnana@uhs.ae; 5 Clinical Sciences Department, College of Medicine, University of Sharjah, Sharjah 27272, UAE

**Keywords:** hypoglycemia unawareness, type 1 diabetes mellitus, cytokines, inflammatory markers, TNF-α, IL-1β, IL-6, IFN-γ

## Abstract

The recurrence of hypoglycemic episodes leads to attenuation of the normal counter-regulatory mechanisms that are controlled by the hypothalamus, which results in hypoglycemia unawareness (HU). In this case report, we described for the first time the differential expression of TNF-α, IL-1β, IL-6, and IFN-γ in a blood sample that was taken from a 27-year-old patient with type 1 diabetes mellitus (T1DM) who was diagnosed with HU. The anti-diabetic regimen is currently based on insulin injection, but the patient is planning to start the use of an insulin pump to have better control of glucose levels. Our results showed a trend toward an increase in the expression of IL-1β, IL-6, and IFN-γ in T1DM patient with HU. However, the mRNA level of TNF-α showed a significant decrease. These observations suggest that systemic inflammation could be an underlying cause of HU.

## 1. Introduction

Glucose is the main energy source for the brain which is used for facilitation of several crucial processes such as cognitive functions [1,2]. Low levels of glucose trigger the activation of a counter-regulatory response (CRR) which mainly involves the release of epinephrine [3]. The secretion of epinephrine from adrenal medulla is mediated by the sympathetic nervous system after sensitization of the glucose-sensing neurons in the hypothalamus [4,5,6].

Recurrent experience of iatrogenic hypoglycemic episodes leads to disturbance of the CRR, and this has been known as hypoglycemia-associated autonomic failure (HAAF) [5,7,8]. The latter is mainly caused by the use of anti-diabetic medications, in particular, insulin-based treatments, in patients with type 1 DM (TIDM) [9]. The main outcome of malfunction of CCR is the blunted response of epinephrine to hypoglycemia and this in turn leads to unawareness of hypoglycemia [10]. Hypoglycemia Unawareness (HU) is a condition in which asymptomatic hypoglycemia occurs, due to the absence of the autonomic warning symptoms in response to hypoglycemia [7]. Although recurrent hypoglycemic episodes have been known as the leading cause of HAAF, the mechanisms are not clearly established. We suggest that the low level of glucose is a causative candidate of neuroinflammation and its adverse effects on the survival of hypothalamic neurons which eventually leads to HU. Previous studies have supported this hypothesis by showing that low levels of glucose elicited inflammatory responses in people with diabetes [11]. Here, we report on a case of a T1DM patient who has been diagnosed with HU, and we aim to investigate the relationship, if any, between inflammation and HU.

## 2. Case Description

### 2.1. Hypoglycemia Unawareness (HU) Patient

A 27-year-old woman who had been diagnosed with T1DM for eight years, suffered from recurrent severe hypoglycemia and HU since 2014. The patient was presented to the clinic with a body weight of 56 kg and a body mass index (BMI) of 22.43 kg/m^2^. The latest fasting glucose and HbA1c of the patient were found to be 258 mg/dl and 7.8%, respectively. She experiences asymptomatic hypoglycemia frequently at night and when skipping lunch. Besides her diagnosis with HU, she had been diagnosed with Vitamin D deficiency two years ago. Due to continuous use of insulin injections, she has mild lipohypertrophy on her arms and thighs. No family history related to the current conditions has been reported.

### 2.2. Current and Future Therapeutic Interventions

The patient is currently on a combination of ultralong-acting insulin and fast-acting insulin regimen. She is having 22 U/day of insulin degludec, taken at night, and a total of 36 U/day of insulin lispro, divided into three doses, 10 U before breakfast, 12 U with lunch, and 14 U with dinner. In addition, she is having 1 tablet/day of 50,000 U cholecalciferol for her Vitamin D deficiency. Future management plans include the use of an insulin pump for a better glycemic control.

### 2.3. Blood Samples Collection and Inflammatory Biomarkers Assessment

The Blood sample of 5 mL was collected from the HU patient and other two subjects: a 30-year-old T1DM patient with a BMI of 21.16 kg/m^2^, and a 24-year-old healthy person with no diabetes or any other diseases. The present study was conducted at the University Hospital Sharjah (UHS), Dubai Hospital (DH) and Sharjah Institute for Medical Research (University of Sharjah, UOS). The approval of the study was obtained from the ethics committee of UHS (UHSREC042018, April 2018), DH (DSREC-09/2018_13, October 2018), and UOS (REC-17-08-0801, November 2017) and conducted in accordance with the Declaration of Helsinki. All participants were asked to sign an informed-consent form written in their native language.

Total RNA was extracted from whole blood using QIAamp RNA Blood Mini Kit (Qiagen, Hilden, Germany) following the manufacturer’s protocol. The isolated RNA was then quantified by the Nanodrop2000 spectrophotometer (Thermo Fisher Scientific, Waltham, MA, USA), and purity was determined by the A260/A280 ratio. The RNA was then reverse transcribed to 1000 ng/mL cDNA using the High Capacity cDNA synthesis kit (Applied Biosystems, Foster City, CA, USA).

The quantitative real-time PCR (qRT-PCR) experiments for measuring gene expression of the proinflammatory cytokines were performed using the QuantStudio 3 Real-Time PCR (Applied Biosystems, Foster City, CA, USA) system with a total reaction volume of 10 µL containing 5 μL of 1× Power SYBR green master mix (Applied Biosystems, Foster City, CA, USA), 1 µL of 10 μM forward and reverse primers (Table 1), 1 µL of NFW, and 2 µL of cDNA. The cycling parameters included initialization at 95 °C for 2 min followed by denaturation at 95 °C for 15 s, then annealing at 60 °C for 1 min, and extension for 60 °C for 1 min for a total of 40 cycles. Relative gene expression was determined using the 2^(−∆∆Ct)^ method, and Glyceraldehyde 3-phosphate dehydrogenase (GAPDH) was used as the house keeping gene. The human primer sequences used for the specific amplification of TNF-α, IL-1β, IL-6, IFN-γ, and GAPDH are listed in Table 1. Samples implemented with NFW instead of cDNA were regarded as negative controls.

### 2.4. Gene Expression of Inflammatory Markers in T1DM and HU

**Figure 1:**
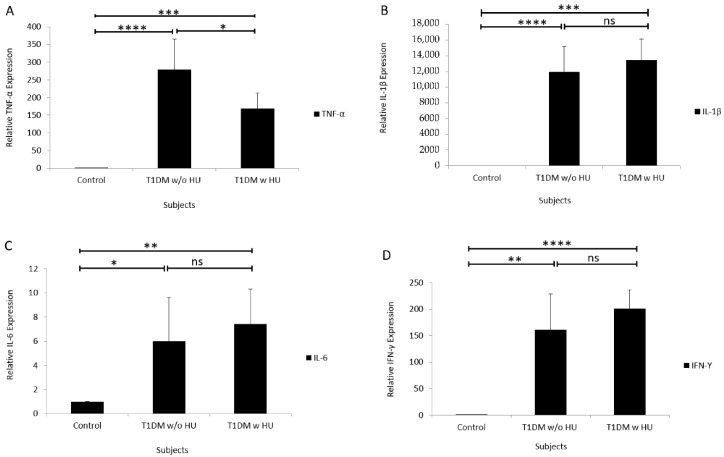
Differential gene expression of inflammatory markers in patients with TIDM with and without HU. (**A, B, C**, and **D**) show expression of TNF-α, IL-1β, IL-6, and IFN-γ in healthy, TIDM without and TIDM with HU, respectively (**** *p* < 0.0001, *** *p* < 0.001, ** *p* < 0.01, * *p* < 0.05). Abbreviations; HU, hypoglycemia unawareness; ns, not statistically significant; w/o HU, without hypoglycemia unawareness; w HU, with hypoglycemia unawareness.

In the qRT-PCR experiments, we aimed to compare the mRNA expression of four cytokines in the blood samples of T1DM patient with HU, T1DM patient without HU, and healthy subject. As represented in Figure 1A, there was a significant increase in TNF-α expression in the patient with T1DM without HU as well as in the patient with T1DM with HU compared to healthy subject (control), whereas TNF-α expression was significantly higher in T1DM without HU compared to T1DM with HU.

Unlike TNF-α, there was an increase in the expression of IL-1β in the patient with T1DM and HU compared to the patient with T1DM without HU; however, this difference was not statistically significant (Figure 1B). Expression of IL-1β in both patients with TIDM, with and without HU, was significantly higher compared to control.

A similar pattern was observed in the expression of IL-6, which was significantly higher in the patients with T1DM with or without HU compared to control. In addition, an increasing trend was observed in the expression of IL-6 in the patients with HU compared to the patients without HU, but this result was not statistically significant (Figure 1C).

As illustrated in Figure 1D, a significant increase in the expression of IFN-γ in the patients with T1DM with and without HU was demonstrated compared to healthy control. Although there was an increasing trend in the expression of IFN-γ in the patients with TIDM and HU compared to the patients with T1DM without HU, this increase was not statistically significant.

## 3. Discussion

HU is a very challenging medical condition in which patients lose their ability to recognize hypoglycemic episodes, and this increases the risk of having serious outcomes such as coma [12]. The pathogenesis of HU is yet to be elucidated; however, previous studies have suggested a link between hypoglycemia and inflammation [12,13,14]. The present report aimed to investigate the relationship, if any, between HU and systemic inflammation. We have demonstrated that the TNF-α mRNA level in the T1DM patient with HU was about half of that in the patient without HU. This finding may contradict with some studies that showed an increase in TNF-α plasma levels during hypoglycemia in T1DM patients compared to patients who were euglycemic [15]. However, this could be explained by the fact that the present report includes only one case of HU. In addition, previous reports have investigated only hypoglycemia cases which are different from HU. The latter involves recurrent episodes of hypoglycemia, which leads to manipulation of the hypothalamic neural function. On the other hand, previous studies have shown that neurological disorders exhibit different functional roles of TNF-α compared to other cytokines reflecting a distinct impact of TNF-α on the brain networking. It was found that a longer duration of the expression of TNF-α is necessary for producing adverse effects of this cytokine on neurons. This may provide an alternative explanation for the differential expression of TNF-α in our report [16].

IL-1β mRNA in the blood sample of the T1DM patient with HU showed a higher level than the T1DM patient without HU. This may be consistent with a study by Nematollahi et al. which showed an increase in the IL-1β level after insulin-induced hypoglycemia in healthy subjects [12]. Moreover, the mRNA expression level of IL-6 was also higher in the T1DM patient with an impaired awareness of hypoglycemia than the patient who is aware of hypoglycemia. IL-6 is one of the most commonly studied cytokines for the correlation with hypoglycemia. It was shown in previous studies that high IL-6 plasma level is associated with frequent hypoglycemia episodes [17].

Lastly, investigation of IFN-γ is considered as one of the novel aspects of the present study as no previous studies included this cytokine in relation to HU. The present report has revealed that IFN-γ mRNA levels in the patient with HU increased compared to the patient without HU, however, it was not statistically significant. Although there are no current studies indicating any relationship between hypoglycemia and IFN-γ, it was found that IFN-γ disrupts the brain’s immune cells in the diabetic brain [18]. Interestingly, more recent studies have suggested that IFN-γ represents a link between diabetes and neurodegenerative disorders such as dementia [19]; therefore, it is of great interest to include IFN-γ in the present study and in our future study.

The number of subjects in our report can be considered as the main limitation of this study. Among all the recruited T1DM patients, only one patient was found to have confirmed HU. This can be improved in future studies by increasing the sample size using a multi-centered approach.

The results of this case report suggest a relationship between HU and inflammation given the hypothesis that the latter is the underlying cause of HU. Our results revealed a trend toward an increase in the expression of IL-1β, IL-6, and IFN-γ in the T1DM patient with HU. However, the mRNA level of TNF-α showed a significant decrease. These observations suggest that systemic inflammation could be an underlying cause of HU. Therefore, future well-powered and rigorous controlled studies are needed to further evaluate the role of hypothalamic inflammation in the HU pathophysiology.

## Figures and Tables

**Table 1:** Human primers used for quantitative real-time polymerase chain reaction.

Gene	Forward Primer (5′–3′)	Reverse Primer (5′–3′)	Accession No.
**TNF-α**	CTCTTCTGCCTGCTGCACTTTG	ATGGGCTACAGGCTTGTCACTC	NM_000594
**IL-1β**	CCACAGACCTTCCAGGAGAATG	GTGCAGTTCAGTGATCGTACAGG	NM_000576
**IL-6**	AGACAGCCACTCACCTCTTCAG	TTCTGCCAGTGCCTCTTTGCTG	NM_000600
**IFN-γ**	GAGTGTGGAGACCATCAAGGAAG	TGCTTTGCGTTGGACATTCAAGTC	NM_000619
**GAPDH**	GAAATCCCATCACCATCTTCCAGG	GAGCCCCAGCCTTCTCCATG	NM_002046

## Data Availability

The data presented in this study are available on request from the corresponding author.
